# Integrating parent voices into research at the extremes of prematurity: what are we doing and where should we go?

**DOI:** 10.1038/s41372-024-02165-1

**Published:** 2024-11-15

**Authors:** Katharine P. Callahan, Anup C. Katheria, Thuy Mai Luu, Rebecca Pearce, Annie Janvier

**Affiliations:** 1 Division of Neonatology, Children’s Hospital of Philadelphia, Philadelphia, PA USA; 2 Department of Medical Ethics and Health Policy, The Perelman School of Medicine at the University of Pennsylvania, Philadelphia, PA USA; 3 The Perelman School of Medicine at the University of Pennsylvania, Philadelphia, PA USA; 4 Sharp Mary Birch Hospital for Women and Newborns, San Diego, CA USA; 5 Department of Pediatrics, Université de Montréal, Montréal, CA Canada; 6 Research Center, CHU Sainte-Justine, Montréal, CA Canada; 7 Parent Representative: CHU Sainte-Justine and Canadian Premature Baby Foundation, Montréal, CA Canada; 8 Division of Neonatology, CHU Sainte-Justine, Montréal, CA Canada; 9 Bureau de L’éthique Clinique, Université de Montréal, Montréal, CA Canada; 10 Unité D’éthique Clinique, Centre D’excellence en éthique Clinique CHU Sainte-Justine, Montréal, CA Canada

**Keywords:** Outcomes research, Ethics

## Abstract

When a baby is born premature, a landscape of potential problems replaces an imagined future. Outcomes become the measures of success. Researchers are recognizing that we need the direct input of parents to select meaningful outcomes. In this article, we describe how researchers and clinicians in neonatology have historically defined outcomes and the limitations of these methods. We chart the integration of stakeholders—patients and parents—into outcomes selection. ‘Parent-important outcomes’ are those deemed most important by parents, as the voices of their children. We outline a path toward determining parent-important outcomes in neonatology through mixed methods research. We conclude by suggesting how parent-important outcomes can be integrated into neonatal follow up research and clinical trial design. Ultimately, all researchers of prematurity aim in some way to improve outcomes that parents and patients care about. We hope this article will remind us of this beacon.

## Outcomes of prematurity

During pregnancy, most prospective parents hope for healthy babies who will develop progressive independence and lead fulfilled lives. If things go well, parents are unlikely to define these hopes as “outcomes.” When a child is born extremely premature, however, this imagined future is replaced with a landscape of potential complications and deviations. At earlier gestational ages, complications are both more likely and more likely to be severe. Some children will die, and others will survive with different outcomes, categorized by the medical system as either “good” or “bad.” Neonatal databases record adverse outcomes in the neonatal intensive care unit (NICU) such as bronchopulmonary dysplasia (BPD), retinopathy or prematurity, and infections. Neonatal follow-up clinics examine and test children to categorize them as having mild, moderate, or severe neurodevelopmental impairment.

All these classifications and categorizations—the markers of success—have been chosen by clinicians and researchers, without parental input. Outcomes orient researchers and determine the targets of quality improvement work. They are also the crux of treatment decisions, sometimes determining goals of care. We need the direct input of parents to select meaningful outcomes. In this article, we will describe how researchers and clinicians in neonatology have historically decided on the outcomes to be measured after preterm birth, the limitations of current measures, and a path toward re-centering outcomes around families.

## A parent’s story—written by Rebecca Pearce, mother of Maren and Lily

One of my earliest memories of my daughter’s 4-month NICU stay 15 years ago is standing in her hospital room and peering at an X-ray of her cloudy lungs. It was several weeks after her birth, and a kind neonatology fellow was trying to explain to us that the X-ray, and her continued respiratory support, indicated that Maren was developing bronchopulmonary dysplasia, or BPD. I later learned this meant oxygen need at 36 weeks. She had been born at 25 weeks and 5 days gestational age, and the vicious cycle of lung damage from necessary respiratory support meant she needed more respiratory support which resulted in more lung damage. I remember asking if maybe her lungs would clear up, and the fellow kindly but skeptically said that it was unlikely. He clearly didn’t want to crush me, but I’m sure he had seen enough of these X-rays to know exactly how things were going to go.

The term BPD and its associated levels—mild, moderate, or severe—meant nothing to me and my partner. We both worked in scientific domains, but stats did not mean much anymore. We had twins, but 50% had died. We were scared for Maren. What did mean something, in the worst possible way, was Maren’s respiratory instability, the seven weeks she spent on a ventilator, her terrifying desaturations, the awful disappointment of having to go back and forth between CPAP and an oxygen cannula, and the terrible stress of waiting every few days to see whether her blood carbon dioxide levels were low enough for her to be released from the hospital so we could finally go home and try to be a regular family. To the kind fellow, BPD seemed to be an end point. But to me it was a code, connecting our terrifying present to an unknown future. What I wanted to know was whether I would be able to take Maren home and how I would manage having an oxygen condenser installed in my house. How often I would need to come to the hospital, what life was like for other families. What I wanted to know was whether I would ever be able to return to work or take Maren to a birthday party with other children, if she would ever be able to exercise like other children.

As an outcome of Maren’s prematurity, a diagnosis of BPD had unclear significance. Its relationship to the life I hoped for, for Maren and myself, was as hazy as her lungs. Ultimately, I took two years’ leave from my job, rather than the year we had planned, so that we could wait as long as possible for Maren to attend daycare where she would be exposed to dozens of respiratory viruses. While I was fortunate to be able to do this, it had financial and professional impacts. Maren was hospitalized multiple times with pneumonia before she was five. I remember the feeling of dread and helplessness when she would start to cough, then wheeze, and we knew it was time for another visit to the ER where we would spend days confined to a hospital room with a toddler attached to an IV. Maren developed asthma and had to use two different types of inhalers for years, as well as have visits to the pulmonologist every 6 months. We had to carefully consider how we socialized. Activities like swimming or playdates, what should have been joyful moments, generated risk and uncertainty, becoming a kind of virus Russian roulette – would Maren get sick or not?

What we considered to be important outcomes related to breathing were rehospitalizations, visits to medical specialists, negative impacts on stress levels, isolation, socialization, daily activities, and quality of life. Research data align poorly with the questions we had, staring at Maren’s hazy film and following discharge. For other families, children might have serious feeding or sleeping problems linked to breathing, but these are also rarely measured. Parents care about function, participation, and quality of life over labels. Ultimately, medical interventions should aim to improve what the child will be able to do on a daily basis at home, at school or in society, in light of their particular limitations and strengths.

## Limitations of currently measured outcomes

Defining outcomes is challenging in all of medicine, but for at least five reasons it is particularly difficult for infants born extremely premature. First, extreme prematurity, and the neonatal intensive care required to survive it, affects a child’s entire body [1]. Defining outcomes that represent the whole, or distinguishing meaningful components, is difficult [2]. Second, infants do not have a prior baseline with which their parents or clinicians can compare their outcomes. Outcomes are instead relative to a hypothetical alternative course, a term birth, making them more abstract. Third, even in the best of circumstances infants cannot function independently, so most outcomes in infancy will, in a sense, be surrogate outcomes, intended to predict future adult outcomes. Many outcomes of ultimate significance relate to childhood or adulthood and will not be apparent for years or decades. Fourth, babies’ outcomes will impact their whole families, in positive and negative ways, which can be difficult to measure. Finally, the rarity of extreme prematurity renders accruing sufficient data to draw meaningful outcomes very difficult. Extremely preterm infants for available study are (thankfully) a rare resource, which must be used carefully toward the goal of improving the lives of these children and families.

Historically, in neonatology, as in most fields of medicine, parents have not played a central role in selecting outcomes to collect during the NICU hospitalization, at neonatal follow-up, or during clinical trials. Researchers and clinicians have been the ones who defined success. This has produced outcomes that are increasingly recognized to correlate poorly with parents’ priorities. We emphasize the fitness of one’s body—such as growth measures and Bayley scores [3]—over functional assessments of abilities and disabilities, as the result of history [4] and our own ableism [5–7]. Neurodevelopment is in many studies considered the sole marker of success. We dedicate enormous resources to gather data which may predict neurodevelopment, such as prognostic MRIs, that parents may not find important [8]. The ‘F words’ is a model of childhood disability that highlights many domains missing from traditional NICU outcomes. In this model, fitness of a child (individual growth and development) is on equal ground with function, friends, family, and fun [9, 10]. Indeed, NICU parents report that how a child can engage in activities (like eating, walking, communicating) and participate in daily life (e.g. having friends, going to school) are hugely important. And how this occurs within a social context (i.e. the family,) and based on the individual’s personal attributes (i.e. behavior, temperament) also deserve attention [11].

Methods for recording outcomes that have been employed historically are often not in line with parents’ experiences. We have arbitrarily dichotomized continuous outcomes [2, 12]. We have grouped outcomes of clearly unequal significance to parents, such as death and BPD [13]. We have also combined outcomes in ways that may not meaningfully represent a child’s overall status, such as in quality of life measures [5, 14, 15]. Our overall assessments of patients’ neurodevelopment—mild, moderate, severe—correlate poorly with parents’ assessments, who generally disagree with these classifications [16]. Our outcome measures produce results that are more negative than the assessments of parents or even ultimately adults who were born preterm [11, 17]. In sum, research in neonatology uses outcomes of questionable importance to parents and therefore often produces data that are difficult to apply to clinical practice.

Measuring outcomes that are misaligned with parents’ priorities can be harmful in several ways. First, clinicians and parents often face tradeoffs, and they will be unable to effectively weigh outcomes for which there is insufficient data. Consider the example of steroids for lung disease of prematurity. We know that steroids help with short-term respiratory outcomes [18]. However, they may have a negative impact on lung growth, perhaps due to decreased inflammation, which may worsen outcomes parents care about such as long-term daily functioning [19, 20]. We need to know precisely what long-term outcomes are most important to parents, so that we can collect targeted data that inform tradeoffs of steroid use. Second, outcomes are used to target resources. For example, cerebral palsy gives a named diagnosis to a constellation of neurological symptoms and carrying such a diagnosis entitles patients to defined resources. Other unnamed outcomes that may be equally important to families, such as issues with feeding or impact on siblings, have garnered less attention as research outcomes and therefore are not connected to resources in the same ways.

In sum, to collect clinically useful data and improve neonatal care, researchers need to know what parents see as most important.

## Terminology in centering outcomes around patients

For thousands of years, medicine evolved as an authoritarian practice; ‘the doctor knows best’ [21, 22]. Since the end of the 20^th^ century, patients have increasingly been seen as participants in, rather than just recipients of, medical care.

A number of terms have emerged to describe the relationships between patients and outcomes, and clarifying the meaning of these terms is necessary for considering patients’ role in outcome prioritization. Because neonates are not good at reporting their perspectives, parents (or other guardians) are accepted as their proxies, and the terms must be adapted accordingly. One focus of research has been on communicating expected outcomes to patients [23, 24]. Outcome data are certain to be ineffective if parents cannot understand it. Related to this focus, the term ‘patient-important outcomes’ has been defined as “a characteristic or variable that reflects how a patient feels, functions or survives” [25]. In contrast to ‘biochemical outcomes,’ such as lab or imaging findings, or ‘surrogate outcomes,’ that are intermediate end points, ‘patient-important outcomes’ are perceptible within humans’ lived experience. This term has been useful in demonstrating that many studies use outcomes not even perceptible to patients [26]. In neonatology, for example, periventricular leukomalacia or perhaps even BPD, are surrogate outcomes that have been extensively studied but are of poor predictive value for outcomes a patient, or parent, would perceive [6, 27–30]. However, though ‘patient-important outcomes’ are perceptible to patients, they are not necessarily *prioritized* by patients.

Another parallel body of work has emerged on ‘patient-reported outcomes,’ defined as “any report coming directly from patients… about the status of a patient’s health condition without amendment or interpretation” [31]. Measures directly from patients are increasingly recognized to be reliable, valid study metrics [32, 33], which facilitates studying real-world impacts of medical problems that were previously difficult to capture. Still, the outcomes that patients are asked to report on are frequently those prioritized by physicians and researchers.

At the intersection of these terms arises the concept of ‘patient-centered outcomes,’ defined as outcomes meaningful and prioritized by patients [34]. Patient-centered outcomes provide the answers patients most want when they ask ‘what will happen?’ or ‘what will this be like?’ Parallel to this concept emerges the idea of outcomes prioritized by parents, as the voices of their children. Terming such outcomes ‘parent-centered outcomes’ would be most concordant with the meanings of other terms we have reviewed here but has the unanticipated effect of suggesting a focus on parents over their children. We therefore suggest the term ‘parent-important outcomes’ to signify outcomes that are *most* important for children, as indicated by their parents (Table 1). We use “parent” in the most inclusive sense, indicating all caregivers who have a stake in a child’s outcome, and ultimately even the children themselves, engaged as feasible. The remainder of this article will be dedicated to considering how we can: (1) engage parents to determine parent-important outcomes and (2) translate parents’ priorities into valid research measures.

**Table 1:** Questions to consider in implementing parent-important outcomes.

**Reading a research paper:** • How were these outcomes chosen? (and who chose them?) • What is their relationship to clinical practice and what might parents perceive and prioritize?
**Designing a research project:** • What is known about parent-important outcomes in this field? • What metrics exist or could be created to measure these outcomes? • How can I directly involve parents in the design, implementation, and analysis for this study?
**Talking to parents:** • What does this parent in particular seem to care about? You can ask! • How can I speak about available information in ways that will align with their priorities? • How can I incorporate the F words model (fitness, function, friends, family, fun, fitness) to provide parents with more holistic information? [9]

## Engaging parents to determine parent-important outcomes

To determine what outcomes are most important to parents, researchers need to consider all potential outcomes, being open minded about what constitutes an ‘outcome.’ The story of Maren and Lily, told by Rebecca, is the story of one parent. The aim when examining parent-important outcomes is to know what is important to many/most families and where families may disagree.

We need to begin with open-ended questions, for two reasons: (1) in order to avoid validating “medical-important outcomes” with parents and (2) to enable detection of outcomes not typically measured in medicine. Asking parents to rank BPD, necrotizing enterocolitis, and retinopathy of prematurity in terms of severity, for example, presupposes that these medically defined outcomes are conceptualized in ways meaningful to a parent. They might not be. Instead, we could ask parents what outcomes related to breathing or eating are important to them, which may reveal different ways conceptualizing problems with these organ systems. For example, in ranking twenty possible breathing outcomes, parents rank ‘feeling like my child is safe at home’ to be the most important breathing-related outcome of prematurity. What enables the parent of a child with breathing problems related to prematurity to parent to feel safe at home? Addressing this question will lead us to very different approaches than aiming to reduce BPD. Perhaps also we need to investigate medical other outcomes then “BPD? What are medical outcomes that relate with parent important outcomes?

## The ‘Parents’ Voice Project’ in neonatology

The Parents’ Voice Project set the stage for determining parent-important outcomes in extreme prematurity. Over one year, we approached all parents of children born at less than 29 weeks in a neonatal follow-up clinic in Canada and recruited parents of over 1000 children. In the first stage, parents were informed that doctors would sort their child into one of 4 groups during their visit: no, mild, moderate, or severe neurodevelopmental impairment. They were also asked how they would classify their child. There was only fair agreement between parent-reported and the medical classification; families usually viewed their child as less impaired [35]. Similarly, in a second portion of the Parents’ Voice Project that employed hypothetical vignettes, parents reported that many health states commonly considered severe by researchers were not severe in their mind. For example, having hearing impairment requiring cochlear implants or language delay were considered severe only by 17% of parents [16]. In a final stage of the Parents’ Voice Project, we asked a total of 248 parents to help identify relevant outcomes that should be communicated to parents in the NICU. We questioned them on the impact of the preterm birth on their lives, the health of their child, and their information needs [36, 37]. Parents reported outcomes that related to function (what the child could do, e.g. breathing, walking, talking, eating) rather than specific diagnoses (e.g. cerebral palsy) [38]. The majority identified positive and negative impacts after preterm birth [37]. Parents wanted a more balanced perspective, more optimism from doctors, and more practical advice [39].

## Parents are all different

Not all parents will agree about what outcomes are most important [40, 41]. Therefore, capturing diverse and varied parent perspectives and sampling parents at different time points with respect to their NICU admission are essential. High participation rates are essential to ensuring generalizability, and, in particular, to eliciting the voices of parents from groups historically neglected in medical research [42]. Engaging parents of critically ill neonates or young children with medical complexity in research is inherently difficult. Consequently, researchers need to make it as easy as possible for parents to participate. Providing in-person and virtual options, enabling participation in the early morning or late at night, providing support for non-English speakers, and fairly compensating parents for their time and effort can help [37, 42, 43].

Researchers need to directly evaluate the ways in which confounding variables, such as demographic and social factors, correlate with perspective. Only by understanding variations in parental perspectives can we tailor interventions in the future. For example, parents with limited parental leave may ascribe particular importance to readmission or daycare absence. Directed advocacy might aim to study the medical impact and cost efficacy of additional leave time for parents. Parent perspectives are also likely to change as children age, their illness improves or progresses, and parents gain experience [44, 45]. For many studies which aim to improve the long-term health of babies born extremely preterm, perspectives of parents at the most distant timepoint feasible may be most important. Yet, some studies may aim to improve the NICU experience itself, in which case the perspective of parents currently in the NICU may be optimal.

Aiming for consensus, through Delphi or other methods, is problematic because variation in parent-importance needs to be investigated rather than overlooked.

## Ranking outcomes of importance

While many outcomes may be important, determining the relative importance of numerous outcomes for groups of parents is essential to research prioritization. Likert scales and ranking exercises have been employed for this purpose in other fields, such as ranking outcomes of importance in psoriasis treatments [46]. Another well-validated method for determining the relative importance of multiple attributes is discrete choice experiments (DCE) [47]. This method enables discrimination between many outcomes, all of which are expected to be important. DCE displays numerous small sets of outcomes and has respondents rank the most and least important within each set, ultimately producing scaled importance scores for each outcome [6, 47, 48]. Discrete choice experiments can also ask parents to compare combinations of outcomes, capturing real life tradeoffs.

Whatever the method, researchers will need to evaluate severity thresholds within outcomes as well as the relative importance of different outcomes. For instance, a parent is likely to consider their child requiring respiratory support to be a severe outcome. But determining the level and duration of respiratory support required to designate the outcome as severe is unknown.

## Integrating parent-important outcomes in neonatal follow-up networks

There are many ways in which parent-important outcomes, once known, can be integrated into research, and subsequently clinical care. The most straightforward way is to prioritize validated metrics on topics parents care about. For example, following the Parents’ Voice Project, the Canadian Neonatal Follow-Up Network (CNFUN) completed a reevaluation of all outcomes measured.

Like many countries, Canada has a system for collecting outcome data at follow-up on all extremely preterm babies and harmonizing data across centers using a shared database. Historically, these databases include data primarily on individual fitness without consideration of the other F-words: function, friends, family, and fun. Databases, therefore, have not captured outcomes holistically. As part of the Parents’ Voice Project, results from all studies were reviewed with a group of NICU parents, neonatal follow-up clinicians, and researchers. The group agreed on a set of priorities: child well-being, child function/quality of life, socioemotional and behavioral outcomes, respiratory health, feeding, sleeping, and caregiver well-being and mental health, which map with the ‘F words’ [49]. Working groups identified instruments, mostly validated parent-reported questionnaires, or items that could be found through chart review or medical interview to measure in the most efficient manner these outcomes [50]. To avoid increasing the burden on families and clinicians with too many questionnaires, the group had to make compromises. For example, for each new item added on the case report form, an older item, deemed to be less relevant, was removed. Considerations for feasibility (time, cost, literacy), appropriateness and relevance, both on clinical and research standpoints, were also taken for questionnaires, with the goal of only keeping one or two to increase completeness and accuracy. It was therefore decided to prioritize standardized and validated questionnaires on quality of life and functioning, which were felt to not be covered in Network database. This has always been done with engagement of parents and health care professionals involved in follow-up to increase the chance of successful implementation that is sustainable over time and provides valid research data to improve the overall health of extremely preterm children.

## New trial methodologies integrating parent-important outcomes

Neonatal clinical trials must account for competing outcomes and often incorporate many factors into composite outcomes. As a result, neonatal trials commonly use a primary outcome that is an adverse composite outcome of death or a specific morbidity (e.g., “intraventricular hemorrhage, BPD, or death”; “neurodevelopmental disability or death”). Although these composite outcomes have improved trial feasibility and helped address concerns about competing outcomes, they may obscure data that are meaningful to parents and inappropriately equate outcomes of death and morbidity [51]. Several recent trials from the Neonatal Research Network [52–54], a national trial network, have used such traditional composite outcomes but have not demonstrated differences between groups; one reason for such null findings may be that detecting changes requires a more nuanced and holistic approach, as detailed below, with regard to construction of the primary outcome.

Given that most interventions may affect multiple outcomes, and that parent-important outcomes are rarely singular, we need better ways to integrate multiple outcomes and the relative importance of each. A novel approach being used in adult trials is the “Desirability of Outcome Ranking” (DOOR) method. The mathematical basis for DOOR is well-established [55]. This method has been used to reanalyze obstetrical/neonatal trials [56] and the insights it can provide in obstetric and neonatal trials have been demonstrated in secondary analyses trials.

For example, a trial could use a simple morbidity count of lung, brain, or eye injury in the newborn period to determine whether there is an increased risk of longer-term functional impairment. As mentioned above, while the individual outcomes themselves are poor predictors of functional outcomes, the presence of none vs 1, 2, or 3 of these morbidities has been strongly associated with severe delays, deafness, blindness or death by 5 years of age [57–59]. Instead of an emphasis on a single morbidity, the combined outcome measure is centered on the whole child. These outcomes could be adapted to different subpopulations of parents that have different priorities.

Partial credit analysis can enable researchers to incorporate the relative importance of various outcomes [10]. This approach is analogous to an exam where 100 percent is the most desirable outcome and 0% the least (i.e. death), and partial credit (a score between 2 and 99%) allows parents or other stakeholders to assign weights to the other outcomes. Treatment comparison can be made by comparing mean partial credit scores using these weighted allocations. This ensures that a small change in an important outcome does not get missed due to larger differences in less important outcomes. Ideally, various stakeholders are engaged to determine a valid partial credit analysis.

Ultimately, parent-important outcomes need to be valid, reliable, and feasible research measures. Researchers who design new outcomes will need to follow standard procedures for evaluating their psychometric properties [60, 61]. Neonatal research poses particular challenges in terms of follow-up duration. Often, the outcomes we most want to know are future-based. Unfortunately, following patients for as long as would be required to determine such outcomes is often not feasible. Therefore, particular efforts are required to evaluate which proximal outcomes predict parent-important long-term outcomes. These efforts can capitalize on robust methods that exist for this purpose in neonatology, adapting to predict what parents find important [62]. Obtaining follow up from the most remote time points feasible will still be important whenever possible.

## Ending up where we are going

Integrating parent-important outcomes into certain types of research seems straightforward, at least in theory. If we knew what respiratory outcomes parents cared most about, we could define which factors/interventions/outcomes predict these outcomes, and could target these outcomes for study. For other research, using parent-important outcomes is perhaps less intuitive or feasible. Animal-based lab experiments will need to use physiologic outcomes. Early trials of new medications have to establish safety and dosing parameters. Yet, in the end, all researchers of prematurity aim in some way to improve outcomes that parents and patients care about. Even if a study will not directly use parent-important outcomes, researchers can consider and clarify how ultimately their work hopes to improve outcomes parents care about. Lab researchers could consider which physiologic outcomes for mice best approximate the human outcomes parents prioritize. Pharmacologic researchers can consider what safety issues would be most problematic to parents. If we keep in mind where we are going, we are sure to get there more swiftly.
